# Environmental Risk Assessment in Community Care: A Scoping Review

**DOI:** 10.3390/healthcare12080859

**Published:** 2024-04-19

**Authors:** Maryam Rouhi, Tanya Linden, Douglass Doherty, Sarah J. Prior

**Affiliations:** 1 Tasmanian School of Medicine, University of Tasmania, Burnie, TAS 7320, Australia; maryam.rouhi@utas.edu.au (M.R.); tanya.linden@utas.edu.au (T.L.); 2 Family Based Care Association North West Inc., Burnie, TAS 7320, Australia; ddoherty@familybasedcare.org.au

**Keywords:** risk assessments tool, risk assessments, environment, community health service, community health care

## Abstract

Community care encompasses inherent risks for both clients and healthcare providers. Maintaining a safe environment for the delivery of care services ensures that any risk of unintentional or intentional personal harm is minimised. The aim of this scoping review is to (a) provide an overview of existing knowledge by summarising the current literature and (b) identify gaps pertaining to understanding and managing environmental risk in community care settings. Guided by the Population/Concept/Context approach and PRISMA guidelines, this paper used two questions to answer how a score-based tool for assessing client suitability in community care is developed and how an environmental screening tool assists with reducing risk to community care workers. Literature searches of CINAHL, PubMed (Medline), Web of Science and PsychINFO databases were conducted between September 2023 and November 2023. We included full text articles published from 2018 to 2023. The following four broad areas were identified as key components in the structure of an environmental screening tool: environmental factors, health factors, socioeconomic factors and cultural factors. The results of this review provide valuable information which can be utilised by care organisations to develop and/or refine tools to ensure the safety and wellbeing of workers within the community care sector.

## 1. Introduction

Community care services delivered as part of the care economy have been rapidly expanding to meet the needs of individuals and their families, particularly as the ageing population increases globally. In Australia, 1.46 million people (5.8% of the Australian population) reported a core activity need for assistance—self-care, communication and mobility due to disability, long-term health conditions or the effects of old age [1]. People requiring this type of support are known to use formal and/or informal providers of assistance including government or private organisations, as well as family and friends [1]. Community care can be defined as providing the right level of intervention and support to enable people to achieve maximum independence and control over their own lives [2]. Community care workers act at the community level to provide a range of support services so individuals, despite reductions in capacity, can live with independence in their own homes. They may also provide support with a focus on social participation to enable people to engage with activities to cultivate their intrinsic capacity [3] and assist with activities of daily living.

Community care encompasses inherent risks for both clients and healthcare providers. Situations within community healthcare settings can give rise to challenging conditions, and the data from the Australian Bureau of Statistics (2021–22) reveal that the primary group affected by such incidents is community and personal service workers, accounting for 27% of these occurrences [1].

Maintaining a safe environment for the delivery of care services ensures that any risk of unintentional or intentional personal harm is minimised and that organisations mitigate any financial risk. The Australian Capital Territory (ACT) Government suggests that the key to effective risk management in community services is conversation [4]. Understanding the needs, circumstances and goals of the client will assist in developing strategies and responsibilities to work together safely. Understanding the role of the direct care worker in the community further provides strategies for mitigating risk and reducing harm at the personal and organisational levels. Armstrong-Stassen and Cameron [5] suggest that high workloads, vulnerable families and difficult (complex) clients are issues that impact community care workforce satisfaction and overall retention. They list physical safety and psychological distress as two main risk factors for community care workers leaving their professions. Similarly, in the community social work context, user participation, professional-client relationships and organisational culture are factors predicting behaviour in the community setting and thus risk to both clients and direct care workers [6].

A risk assessment is defined as a formal process for identifying, evaluating and controlling for risks relevant to achieving the goals of services. Three main categories of risk previously identified in community care include the client, the home and service arrangements [7]. Each client will have individual needs in terms of health and social wellbeing. It is important to understand these needs to determine any risk associated with illness or injury, activities of daily living and health habits such as smoking or alcohol consumption. The physical environment of the client may pose risks for community care workers via a range of factors such as faulty electrical equipment, poorly placed furniture, obstructed access, or the presence of pets, unwelcome visitors and complex domestic relationships. Furthermore, working in isolation increases potential work-related risks for community workers. For example, Work Safe Australia [8] highlights additional risks to those working in isolation as being linked to physical and personal safety along with issues linked to limited access to emergency services and exposure to violence.

The overarching community care organisation is responsible for ensuring that their workforce is safe and workplace risks have been managed and mitigated appropriately. Each state in Australia has a policy or guide for work health and safety in the community care context with varying degrees of focus on risk management. SafeWork South Australia suggests that there are safety challenges faced not only by direct care workers but also employers, contractors and clients, and provides guidelines for risk management. Similarly, WorkSafe Queensland (2018) [7] and the Home and Community Care Program in Western Australia (2012) have developed frameworks [9] for guiding the identification, management and reporting of risks and hazards in community settings. However, there is a gap in the academic literature pertaining to understanding and managing environmental risk in community care. Therefore, the aim of this scoping review was to explore academic research to understand the current practice of environmental risk assessment in the community care setting. The two questions guiding this review were:

1. How is a score-based tool for assessing client suitability in community care developed?
2. How does an environmental screening tool assist with reducing risk to community care workers?

## 2. Materials and Methods

Guided by our research question, the aim of this scoping review was to summarise the diverse body of current literature to provide an overview of existing knowledge and identify gaps in the field of study. By assessing the extent of available evidence, it aimed to synthesise and clarify concepts and identify key characteristics or factors related to our concepts.

The objective of this scoping review was guided by the PCC approach [10] and it was to identify and describe the existing evidence on environmental screening tools that assist to reduce risk to community care workers. The PRISMA-ScR checklist was utilised to guide this review, guided by the Joanna Briggs Institute approach [10]. A protocol for this review was not published.

### 2.1. Eligibility Criteria

The criteria for this study are outlined in Table 1. Studies published since 2018 were included to ensure that the articles captured the most recent community care systems and processes. We included a range of study types pertaining to our research questions that had a full text available in English. We focussed on tools that assessed the environment that community care staff work in routinely locally, nationally and internationally.

### 2.2. Search Strategy

A search for English language papers was performed in the following electronic databases; CINAHL, PubMed (Medline), Web of Science and PsychINFO between September 2023 and November 2023. Google Scholar was utilised as an additional search tool.

The following search terms were used: “assessment tools” OR tool OR “tool kit” OR “instrument” OR “screening tool” OR “risk assessment” OR “patient assessment” OR “risk taking behaviour” OR “environment* monitoring” OR “needs assessment” OR “environment* assessment” OR “social service assessment” OR “social assessment” OR “questionnaire” OR “client assessment” OR “patient assessment” AND safety OR “suitability for care” OR harm OR housing OR behaviour OR resistance OR support OR culture OR religion OR engagement OR interpersonal OR “medical history” OR “mental health” OR “access to home” OR communication OR “physical ability” OR “cognitive impairment” OR assistance AND “family based care” OR “community health work*” OR “community networks” OR “community health service*” OR “community service*” OR “community care worker”.

### 2.3. Article Selection

**Figure 1:**
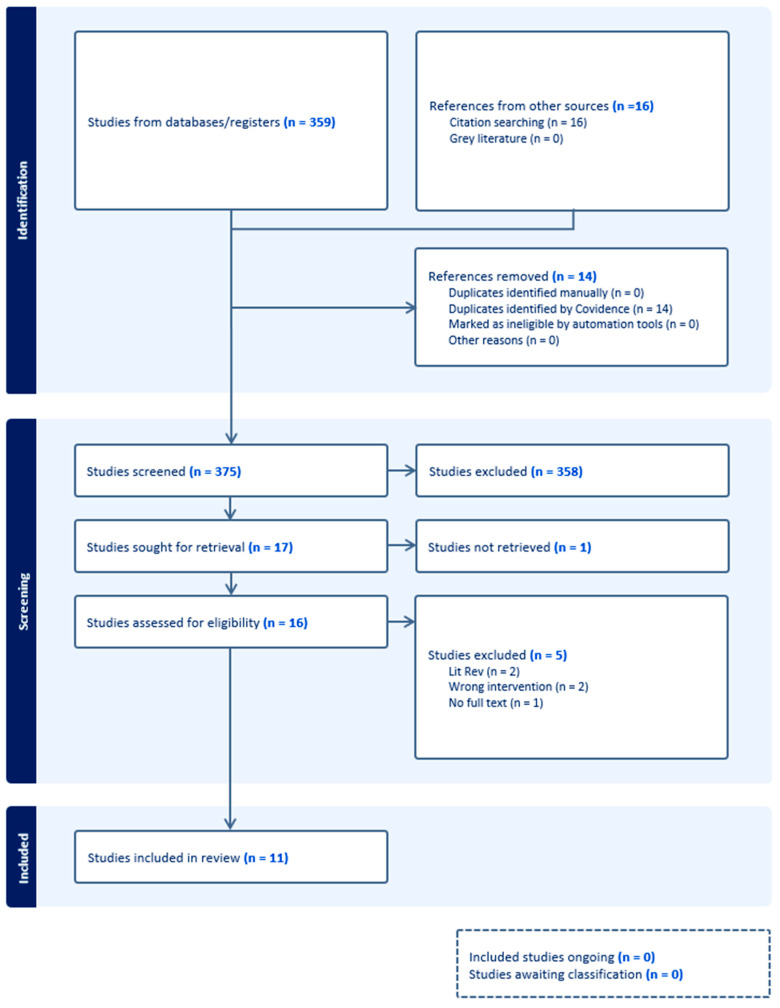
PRISMA scoping review flowchart [11].

Following the search, all records were uploaded into Covidence software [11] and all duplicates were automatically removed. Titles and abstracts were each screened independently by two team members against the inclusion criteria for the review. The full texts of potentially relevant papers were uploaded into Covidence, and full text review was undertaken independently by two team members. Any conflicts were discussed with the whole team (*n* = 3) and a decision was made whether to include or exclude. Reasons for exclusion were recorded and are presented in Figure 1. For this scoping review, the quality appraisal of publications was not assessed consistent with scoping review methodologies [12]. Data were extracted by three team members using a purpose-designed data extraction tool. This included author(s) names, publication date, aims of study, methodology, method, tool, or instrument utilised, country, setting, participants, data analysis and findings (Supplementary Materials).

### 2.4. Charting the Data

The data were initially coded and synthesised dependent on the tools or instruments included in each study and the outcomes from use of these tools. Charting key themes involved scanning and sorting extracted data into these broad categories of tools followed by an inductive analysis aligned with the objectives of this review. All data were sorted in an Excel spreadsheet.

### 2.5. Collating, Summarising and Reporting the Results

The results were organised into a thematic narrative useful for informing the development of a quantitative tool for risk assessment in community care.

## 3. Results

### 3.1. Study Selection

The initial search strategy identified 375 articles. Following screening and selection, 11 studies were included in this review.

### 3.2. Study Characteristics

Eleven papers were included in this scoping review (see Supplementary Materials). The included papers were published in eight different countries: Hong Kong [13,14], Pakistan [15], Japan [16], Canada [17], USA [18,19,20], UK [21], New Zealand [22] and Norway [23]. For this scoping review, six quantitative papers [13,14,16,21,22,23], four qualitative [15,17,19,20] and one mixed method [18] met the inclusion criteria.

Key components of each study were extracted and reviewed by three team members and four factors were utilised to explore the aim of this scoping review in a deductive approach. For the purposes of this review, we have defined these factors as per below.

1. Environmental factors relate to potential hazards in the home describing physical environmental hazards within the layout of the dwelling, its design features and amenities. Environmental factors include such barriers within the physical workspace such as stairs, grab bars and lighting. They also refer to lack of access, inadequate heating/cooling and relate to the environment of the wider community where care and support is provided.
2. Health factors are defined as relating to the mental and physical health and wellbeing of clients, i.e., physical and mental health factors experienced by clients impacting their abilities to undertake daily activities within the home and community.
3. Socioeconomic factors are defined as the social indicators that influence a person’s social standing or class in society. Such factors include social variables related to income, education, place of residence, social inclusion and social barriers.
4. Cultural factors include those factors influencing safety and risk originating from individual belief systems such as religion, as well as race and ethnicity.

#### 3.2.1. Environmental Factors

Four papers outlined a range of environmental factors that may influence the safety of healthcare staff working in the community setting [13,16,18,22].

Lacey and Manuel [22] utilised the standardised Home Care International Residential Assessment Instrument to provide a profile of the sociodemographic, environmental and diagnostic characteristics of older community residents with schizophrenia using a national database. Environmental variables included disrepair of the home, squalid conditions, inadequate heating or cooling, lack of personal safety, limited access to home or rooms in home and limited finances. Nakamura-Thomas and Kyougoku [16] examined the applicability of Comprehensive Environmental Questionnaire for the Elderly (CEQ) for assessing community-dwelling older adults who required support and long-term care (LTC) and support from family members for their community living. They confirmed the secure environment, the interactive environment and the family environment as important environmental factors for elderly people. Mesthrige and Cheung [13] examined environmental factors by categorising them into micro, meso and macro scales for the purposes of investigating ageing in place. The micro-scale described interior design features such as the fit-out of bathrooms, non-slip flooring and grab bars along with increased door width in homes. The meso outlined estate design features highlighting barrier-free access routes, connections between dwellings and other facilities and the macro scale related to the presence of and access to community care support services. Housing, as a specific environmental factor, was assessed in the tool used by Norman et al. [18], who surveyed housing stability, housing type, history of homelessness and the breakdown of the individuals residing in houses where support was provided by community care workers.

#### 3.2.2. Health Factors

Health factors associated with environmental risk were described in five papers included in this study. These factors include diagnostic status of community residents (with schizophrenia) [22], personal experience of illness [21], assessing personal mental health and recovery [23], trauma, [18] and presence of psychosis [19]. Among the included papers, mental health assessment has been recognised most often as a factor contributing to risk and safety issues in the community care sector. Depressive symptoms such as difficulty in sleeping, loss of appetite, reduced concentration, helplessness, suicidal ideation, low energy, difficulty in carrying out social activity, fatigue and agitation contribute to safety concerns for the community care worker as well as the client [15]. Savill et al. found that several client-level factors are associated with resistance to change in services [19]. These factors include suspiciousness, anxiety symptoms, poor general functioning, low functioning, ongoing life stressors and clients’ expressed desire to avoid changing services. Examining psychiatric inpatients’ health status and access to care, including factors such as health status, chronic conditions, body mass index (BMI) and health insurance [18], reveals a comprehensive picture of their wellbeing, alongside insights into the societal services they receive, as indicated by the research of Wilberforce et al. [21].

#### 3.2.3. Socioeconomic Factors

Three papers focused on socioeconomic factors that are linked to safety and risk assessment in the community care setting. Social indicators that contribute to risk in community care were noted as social support, satisfaction with relationships, participation in social activities, community integration, transportation, home-delivered meals, food preparation, personal care (e.g., bathing, toileting, etc., housekeeping, housing assistance, caregiver supports and/or training, financial advice, legal advice and case management [18]. Further, Tan and Chiu [14] proposed that leisure activities, involvement with community groups or organisations, work (for employed participants), opportunity to work (for unemployed participants) and opportunities for contact with family/social inclusion/suitable housing improve outcomes. Social variables have been defined by Lacey et al. [22] as participation in social activities of long-standing interest, visiting a long-standing social acquaintance or family member, conflict or anger with family or friends, being fearful of a family member or close acquaintance or neglected, abused or mistreated. Financial adequacy is another factor proposed by one study [18] and includes employment status (working part-time or full-time), wanting to work but being unable to find a job, and barriers to employment [18].

#### 3.2.4. Cultural Factors

One paper described the potential impact of cultural factors on safety and risk in community care. Abe [20] proposed the concept of a “culture cube”, which was developed to identify and understand the cultural underpinnings of prevention and early intervention work in particular populations. Its aim is to explore cultural beliefs and values and community needs to determine the most appropriate approach to developing interventions. Religion, ethnicity and race were articulated as cultural factors that influence safety and risk in community care in their study.

## 4. Discussion

This scoping review aimed to explore and understand the current practice for environmental risk assessment in community care settings. The two questions guiding this review endeavoured to answer how a score-based tool for assessing client suitability in community care is developed and how an environmental screening tool assists with reducing risk to community care workers, based on four factors. The Australian Commission on Safety and Quality in Healthcare highlights that risk screening tools support the prevention of harm by examining what could cause harm to community health workers in the course of their duties. Ensuring that workers are not physically or psychologically injured involves identifying hazards that may be present in the undertaking of duties, evaluating the level of the risks involved and minimising harm by developing risk mitigating strategies. The results of this review provide important information that can be utilised by care organisations to develop tools to ensure the safety and wellbeing of workers within the community care sector. The current practice evidenced from the academic papers reviewed demonstrates a variety of approaches that are used worldwide when naming and evaluating hazards to ensure safe working environments for community workers.

Environmental risk factors such as housing and access play a major role in the ability of community care workers to complete tasks required for their roles. A study by Rolfe [24] suggests that the role of housing as a social determinant of health and wellbeing for community members is well established, but risk to people working in the community when housing conditions are suboptimal has not been clearly identified. Causal relationships between homes that are damp, contain mould, or other toxins, have inadequate heating or are overcrowded are reported to negatively affect residents’ health [3,25] but there is limited evidence to show how these risks are being considered for healthcare professionals working in these settings.

Similarly, the health and wellbeing of clients in community care have been shown to be risk factors for healthcare workers in this setting [18,21,22,23]. In particular, mental health illness and recovery have been considered as part of social risk screening [26]; however, this is again focused on risk to clients, not healthcare professionals. Understanding the impact of a client’s mental health on the safety and wellbeing of their direct care worker is important to ensure that the provision of care is safe and appropriate for the client, the direct care worker and the healthcare organisation. Mental health is linked to other socioeconomic factors such as social exclusion and poorer access to protective factors (education) [27] that may also play a role in the safety of community care workers.

Socioeconomic factors have been well researched in community care from the perspective of how to improve health and wellbeing of clients. However, the literature assessing risk to community health workers dependent on these types of variables is scarce. This study demonstrated that there are a number of factors and activities linked to socioeconomic status in the community [18,22] but their influence on safety is largely unknown. The deployment of community care workers has been advocated by the World Health Organisation as a key strategy for reaching clients in disadvantaged populations [28]. However, despite evidence that this approach reduces disparities experienced by underserved populations [29], the broad experiences of the community health workers focus more on their own education, acceptance in the community, and career prospects [30], rather than personal safety and environmental hazards or organisational risk reduction.

Cultural differences and the capacity of understanding cultural norms were also raised as potential risks for community care workers in our review [20]. Cultural safety focuses on the needs of the client and the provision of culturally competent and respectful care. However, incorporating these factors into a community care worker risk assessment is beneficial to minimise risk of harm and potentially improve care access for diverse populations.

A small number of the reviewed studies did not assess the performance of screening tools in successfully assisting to reduce risk to community care workers. Those that did noted few barriers to implementation and found tools to be acceptable, feasible, valid and reliable [13,14,15,20,21,23]. Savill et al. (2018) found that using the screening tool did not significantly affect the overall workload of community care workers, suggesting that the benefits from this model would outweigh any time constraints [19].

This convergence of literature from different areas within community-based care identifies several factors which need to be assessed to reduce risk to community care workers. Once identified, work-related safety issues for community care workers can be assessed and strategies put in place to minimise risks. In a sector such as community care, which has high staff turnover [31,32], high levels of stress and burnout [33] and is documented as having higher than average rates of work-related injuries, retaining staff can be difficult. Supporting long-term health and wellbeing of staff and ensuring high-quality care and support for clients play a role in sustainability of the community care model of care.

We anticipate that our results will be of interest to organisations looking to create and/or refine environmental risk assessment tools that broadly screen factors to positively impact the safety of healthcare workers in the community.

### Limitations

Although the literature was searched widely for this review, only 11 studies were found related to the current practices for environmental risk assessments in community care settings. Explanations for the lack of data linked to environmental screening tools may be attributed to the tendency for the development of strategies to screen and assess risk in community care settings to happen at the coal face. Such advances in practice are often not extensively subjected to academic reviews or discussed in academic literature.

## 5. Conclusions

This study has allowed us to assemble strategic data to add to the current understanding of potential factors impacting the safety of workers in community care settings. This review provides a foundation of data to populate an evidence-based environmental screening tool with recommendations relevant to practice, policy and research. Four broad areas identified can be used as key components in the structure of a tool focusing on environmental, health, socioeconomic and cultural factors. Based on our findings, we recommend that further research in this area is imperative to deepen the understanding of factors influencing worker safety in community care settings and to refine the proposed environmental screening tool for enhanced accuracy and effectiveness.

## Figures and Tables

**Table 1:** Eligibility criteria.

Inclusion Criteria	Population	Concept	Context
Published 2018–2023	Community care workers	Risk assessment	International
Quantitative or qualitative	Community health workers	Environmental assessment	Community-based
Case study	Community nurses	Health and safety assessment	
Citation tracking	Support workers	Social assessment	
Mixed methods studies	Allied health staff (community)	Risk reduction	
Full text available		Workplace safety	
		Safety scan	
**Exclusion Criteria**			
Clinical trials	Medical professionals	Clinical assessment	Acute setting
Non-English		Patient care	Hospital-based
Grey literature		Clinical pathway	
Editorials		Telehealth	
Opinion pieces			
Systematic reviews			
Theses			
Technical reports			

## Data Availability

The data presented in this study are available in Supplementary Materials, Table S1.

## Supplementary Materials

The following supporting information can be downloaded at: https://www.mdpi.com/article/10.3390/healthcare12080859/s1, Table S1: Study characteristics. References [13,15,16,17,18,20,22,23] are cited in the Supplementary Materials.
