# Supplementary material for: Environmental Risk Assessment in Community Care: A Scoping Review
Source: Healthcare (Basel). 2024 Apr 19;12(8):859. doi: 10.3390/healthcare12080859 (PMC11050427; doi:10.3390/healthcare12080859)
Supplement: Supplementary file 1 [file healthcare-12-00859-s001.zip › healthcare-2930275-supplementary.pdf]

Table S1. Study characteristics.

Author/s	Abe et.al. (2018)	Biringer et.al. (2018)	Gomez Cardona et al. (2022)	Lacey et.al. (2019)	Mesthrige, & Cheung (2020)	Mohsin et al. (2021)	Norman et al. (2018)	Nakamura-Thomas et al. (2018)	Savill et al. (2018)	Wilberforce et al. (2018)	Tan et al. (2019)
**Aim of study**	To describe the use of a conceptual tool (labelled the culture cube) which was developed to identify and articulate the cultural underpinnings of the PBE approaches within Phase 2 of the California Reducing Disparities Project (CRDP Phase 2), a statewide mental health prevention/early intervention initiative in California.	To translate and adapt the Recovery Assessment Scale-revised (RAS-R) into the Norwegian language and to investigate its psychometric properties in terms of factor structure, convergent and discriminant validity and reliability in the Norwegian context.	To develop a culturally competent and safe tool to assess and promote well-being among the Kanien'kehá:ka people of Quebec	To provide a profile of the sociodemographic, environmental and diagnostic characteristics of older community residents with schizophrenia using a national database.	To investigate the effectiveness of the Ageing in Place facilities design features.	Develop and determine the appropriateness of a culturally adapted tool for assessing maternal depression (CIDT)	to assess the needs of individuals transitioning to the community following psychiatric hospitalization and again 3–5 months later to inform community service planning	This study aimed to identify possible solutions to improve further the Comprehensive Environmental Questionnaire (CEQ) by investigating the way the Likert scale system was being used	to examine providers' experiences of implementing an electronic version of the PQ-B (17) as a screening tool for psychosis.	to design and psychometrically test a new measure of person-centredness to evaluate older people's experiences of community mental health and social care.	To evaluate the reliability of the Mini-SCOPE scale through inter- item consistency and test–retest consistency, as well as the initial correlation with outcome measures of recovery.
**Country**	United States of America	Norway	Canada	New Zealand	Hong Kong	Pakistan	USA	Japan	USA	UK	Hong Kong
**Methodology**	Qualitative	Quantitative	Qualitative	Quantitative	Quantitative	Qualitative	mixed-method	Prospective, cross-sectional quantitative study	A qual	quantitative	adopted a nonexperimental, pre- and post design to validate the psychosocial measurement tool for community services.
**Method**	Initial overview of relevant literature, description of theoretical framework for the culture cube (its development and use) and provision of examples of its application. Followed by brief description of the initial months of the CRDP Phase 2. Finally lessons learned and implications for use of the culture cube to advance our understanding of the cultural underpinnings of practice-based evidence services considered.	A cross-sectional multi-centre study distributed to 231 service users in mental health specialist and community services.	qualitative, collaborative and participatory methods	A cross-sectional study from a continuously recruited national cohort	Questionnaire Likert Scale (face to face)	scoping review and in-depth interviews and focus groups	INTERVIEW/a 2-stage clustered sampling approach where the primary sampling units were hospitals and secondary sampling units were patients.	Likert scale survey	qualitative, semi structured interviews	A postal questionnaire, Likert items	
**Setting**	Community	Community and acute settings	Community	Community-based	Community (Low cost housing)	Community	the primary sampling units were hospitals and secondary sampling units were patients	Community	Investigators recruited participants from screening sites across the Sacramento, California, area. Purposive sampling was adopted, recruiting staff members in a broad range of roles, including clinical, management, administration and support staff.	five English NHS Mental Health Trusts	from psychiatric outpatient clinics

**Participants**		Aimed to include participants representative of all stages of the recovery process. Survey aimed to include ten respondents for each of the 24 items of the RAS-R. Of 322 potential participants, 231 agreed to participate	Twelve adults from health and community services of Kahnawake participated in total five focus group meetings	community-based people aged ⩾65years with an Home Care International Residential Assessment Instrument assessment undertaken between 1 September 2012 and 31 January 2016	N = 224 elderly people living in a new AIP purpose built estate	[mothers, mothers-in-law, and Primary Care Providers (Primary Care Physicians and Lady Health Supervisors N = 55	One thousand one hundred twenty-nine patients from 8 randomly selected hospitals participated.	Aged 60 years and over, living in the community, receiving no rehab services, receiving support from family members, able to participate independently N = 218	Investigators interviewed 17 participants, including two clinical managers, one psychiatrist, six clinicians, one school psychologist, one co-ordinator of student support services, two senior office assistants, one mental health assistant, two chart room managers, and one AmeriCorps volunteer at a community mental health clinic.	596 usable questionnaires were analysed	170 voluntary participants, interview
**Data analysis**	Qualitative	Quantitative analysis	Thematic analysis	Quantitative analysis	1. micro-scale dimension (internal/ interior design features at home), 2. meso-scale dimension (estate design features and social networks), and 3. macro-scale dimension (community care support services)	deductive thematic analysis	Data analysis was conducted using SURVEY procedures in SAS 9.2 (Cary, NC) clustering on hospital, weighted as described above, and applying a finite population correction option to adjust the standard error to account for the limited number (n = 39) of primary sampling units.	Statistical (factor analysis) to examine the validity and reliability of each question (factor) in the CEQ	qualitative analysis	Descriptive statistics	quan analysis

**Findings**	Respecting culture and emphasizing the value and necessity of its inclusion in designing, describing, and evaluating intervention strategies can notably shift the way in which practice-based evidence services (PBEs) are ultimately described and evaluated.<br>If we are to reduce disparities and improve mental health access and outcomes among historically unserved, underserved, and/or inappropriately served communities, then we can and must include, and not dismiss, practices that have “worked” in those communities.	The RAS-R is an acceptable, feasible, valid and reliable tool for assessing mental health recovery, as defined and experienced by service users, in the Norwegian language and context.	Elements central for the sustainability of the initiative include: participatory approach with community members, considering local knowledge and expertise, the values and aspirations of health professionals and organizations, seeking out values and knowledge to help empower and connect the local people to their cultural strengths and healing pathway. Empowerment is a complex strategy that sits within environments and includes sense of community, motivation to act, strong social networks, and resource access. Effective empowerment strategies may depend as much on the leadership of the people involved, as the overall context in which they take place.	Ongoing presence of social and environmental deficits in older age schizophrenia. Individuals with schizophrenia more likely to report negative social interactions and undesirable living conditions. Increased rate of psychiatric comorbidity and diabetes mellitus was also found, while a lower rate of all other physical comorbidities in the group with schizophrenia. Data identified several significant social challenges for people with psychotic illnesses that persist into old age.	General satisfaction with the internal design, comments around good transportation facilities, accessibility, convenient walkways, adequate ramsp, handrails (ability to move freely), flat ground is important, connectivity. Safety is the most important concern	The Community Informant Detection Tool for Maternal Depression (CIDT-MD) is a culturally appropriate, adapted, and an acceptable tool that is easy to understand and use by the community informants in Pakistan.	Demographics (age, race/ethnicity, gender, household income, education, relationship status, children) Housing status (stability of housing, type of housing, history of homelessness, household composition} Health status and access to care (health status, chronic conditions, BMI, health insurances) Social indicators {social support, satisfaction with relationship, activities, community integration) Employment status (working part-time or full-time, wants to work but cannot find a job, Barriers to employment) Other background (criminal justice history, trauma, financial adequacy, spirituality)	Improvements were made the to questionnaire based on the factor loadings in the data analysis. This included changing the wording of the scale ("somewhat was not reliable) and adding descriptors around the scale to provide examples. Additional questions were added to the family environment section to help improve the internal consistency.	Participants reported few barriers to implementation. However, several service-, client-, and program-level factors were considered to significantly affect the implementation of screening. Most participants found that using the screening tool did not significantly affect their overall workload. Facilitators included leadership support, the novelty of using a technology-based screener, regular staff training, and the importance of establishing an effective link between community services and specialty care, with these factors important at different stages of the process. Screening for psychosis was associated with significant advantages over referrals based on clinical judgment alone, including increased speed and accuracy of identification, increased confidence in diagnosis, and the provision of a clear pathway to specialty treatment.	Three factors were identified, representing (i) interpersonal and (ii) organisational aspects of person-centred care; and (iii) negatively phrased items. Removing weaker items resulted in an 18-item scale. The bifactor analysis concluded the summary scale was ‘essentially unidimensional’.	Outcome demonstrated that the Mini-SCOPE scale has good strength of reliability.

**Questions/Instrument Used**	The Culture Cube	Recovery Assessment Scale-revised (RAS-R)	The Growth and Empowerment Measure (GEM)	The Home Care International Residential Assessment Instrument (interRAI-HC). A comprehensive, evidence based standardised assessment tool used by trained health professionals.	Satisfaction with internal design elements (availability of space in home, adequacy and comfort of personal space, ability to continue ADL, sense of self-esteem, sense of belonging, sense of autonomy, privacy level, safety bell and phone, safety measures in home, safety measures in bathroom, satisfaction overall) Perceived importance of internal design features (door size, light switches, non-slip flooring, grab bars, sliding shower head, sunken shower, doorbell push button)	CIDT adapted to assess mothers in the community for depressive symptoms such as Difficulty in Sleeping, Loss of Appetite, Reduced Concentration, Helplessness, Suicidal Ideation, Low Energy , Difficulty in Carrying Out Social Activity, Fatigue, Agitation. A pictorial tool with local examples.	Figure 1, page 4	Secure environment (the first factor) 1. able to feel calm 2. able to receive necessary supports 3. have a comfortable and convenient dwelling 4. has a safe dwelling 5. economically stable 6. able to adequately use medical and welfare services Interactive environment (the second factor) 7. helpful to others 8. has good relationships with friends and acquaintances 9. able to interact with surroundings without difficulties 10. able to go out without difficulties 11. have access to necessary information 12. able to have unlimited external connections Family environment (the third factor) 13. has good family relationships 14. has a living companion	the Prodromal Questionnaire–Brief (PQ-B)	The study comprised the design and implementation of a preliminary 30-item postal questionnaire for self-completion by service users and their families, which through psychometric testing was reduced to a shorter scale with optimised measurement properties.	Variables: leisure activities/ to be involved with community groups/organisations/ work (for employed participants/ to work (for unemployed participants)/ to work (for unemployed participants/ Opportunities for contact with family/ social inclusion/suitable housing/ Increase outcome/
